# Ionization Used in the Treatment of Face Scars

**Published:** 1919

**Authors:** 


***Ionization used in the Treatment of Face Scars.*** By Dr. Hollande. *La Restauration Maxillo-Faciale,* February 1918.

*Technic.* The solution used is a solution of potassium iodide at 1 per cent. The electric current must be feeble, 10 milliamp. at the utmost, the action of potassium iodide upon the scars being more rapid with feeble currents. The positive electrode is usually 5 cm. long and 4 cm. wide. The negative pole is placed upon the scar and the positive one under the chin. The positive pole is soaked with the iodide solution. On an average the treatment is given for 25 minutes daily for one month.

*Clinical Observations.* The scars treated may be classified as :

1. Smooth scars with disparition of the dermis.

2. Fibrous scars with inspissated scar tissues.

3. Scars presenting adhesions to bones.

4. Scars presenting a parchment appearance following generalized burns.

Smooth scars are of a pinky color ; they are flexible and present a certain sensibility to the touch or to thermic influences.

Fibrous scars are like a cord stretched across the skin. They may be several millimeters thick. They seem deprived of all blood circulation. They hinder the movements of the facial muscles and sometimes of the head and neck.

Adhesive scars are generally in the shape of an excavation with a smooth bottom.

Scars with parchment appearance are of a bright copper color. The dermis has disappeared completely. The under-lying muscles can only perform very few and limited movements.

*Treatment.* When applied to smooth scars the process of ionization has the following results : there is an immediate change of color ; the skin passes from purple red to pink, and to a normal condition after a fortnight or three weeks.

Fibrous scars need a longer treatment. They gradually become flexible, but this is only completed after one month at least. Then the scar appears freed from the deep layers of the tissues and has the appearance of normal skin.

With adhesive scars good results were also obtained after one month. The adhesions were completely freed and the superficial layer appeared quite independent of the underlying tissues.

A single case of generalized burn was observed, after having been previously given a treatment with "ambrine". After 6 weeks' treatment the parchment appearance of the skin had markedly disappeared. The skin could be easily wrinkled, the trophic and vascular disturbances had improved after two months and a half, and the condition of the skin was certainly much improved.

60 cases were treated from Nov. 1916, to Dec. 1917.

| | |
|---|---|
| Smooth scars | 10 |
| Adhesive | 6 |
| Fibrous | 43 |
| Scar following generalized burn | 1 |

Out of the 43 cases of fibrous scars, 33 complete successes were obtained. The fair results observed were due to the great size of the cicatricial tissue : scars 15 to 20 cm. long and 2 or 3 cm. wide. Even these very extensive scars could be freed from the underlying tissue after some weeks' treatment; this allowed of their surgical removal and, after a new course of ionization and massages, the scar changed into a very good looking smooth scar.

*Conclusions.* The potassium iodide has a marked influence upon the softening of rigid scar tissue. The scar gradually loses its particular characters : color, stiffness, adhesions. Underlying muscles are freed and the blood circulation becomes normal again.

Adhesions to underlying bones are freed, and trophic disturbances improve.

